# Environmental regulations, green development of agriculture, and residents’ health—empirical analysis of Yangtze River Economic Belt in China

**DOI:** 10.7717/peerj.15717

**Published:** 2023-07-26

**Authors:** Fuling You, Shilong Li

**Affiliations:** 1 School of Management Science and Real Estate, Chongqing University, Chongqing, China; 2 Research Center for Construction Economics and Management, Chongqing University, Chongqing, China

**Keywords:** Environmental regulation, Green development of agriculture, Residents’ health, Empirical analysis

## Abstract

Environmental pollution and food safety have become key public health issues to be addressed in China. Since they are closely related to the green development of agriculture, it is of great practical significance to elucidate the intrinsic relationships between green development of agriculture, environmental regulation and residents’ health. Based on the panel data of the Yangtze River Economic Belt from 2011 to 2020, this study investigates the impacts of environmental regulation and green development of agriculture on residents’ health and the influencing mechanism by applying fixed effects method, mediating effectsmethod and the spatial Dubin method. Results show that the use of chemical fertilizers, pesticides and agricultural films is harmful to residents’ health; environmental regulation has a negative correlation with the green development of agriculture and affect residents’ health through mediating effects; the green development of agriculture has negative spillover effects on residents’ health, indicating that purchasing finished products instead of producing locally reduces the input of production factors such as chemical fertilizers and pesticides and transfers health risks associated with agricultural production activities to neighboring areas. Intensifying command-and-control environmental regulation will induce the expansion of hidden economic activities and harm local residents’ health, while intensifying market-incentive environmental regulation will lead to the ‘Pollution Haven’ phenomenon because of the ‘race to the bottom’, in government and is harmful to the health of residents in neighboring areas. Therefore, it is necessary to formulate reasonable and feasible policies and strengthen the control and prevention of agricultural pollution to enhance green development of agriculture and improve residents’ health.

## Introduction

Agriculture has long been in a fundamental and strategic position in China’s national economy. Over the past 40 years since the reform and opening up, China’s agricultural development has made remarkable achievements, with continuous improvement in agricultural production capacity, significant increase in the output of major agricultural and livestock products, steady increase in farmer’s income, and outstanding contributions to national economy and urbanization. However, China’s agricultural development faces a critical problem: how to feed 22% of the world’s population with 9% of the world’s available arable land  (Zhou, 2002). The sustained increase in the production capacity of agricultural products has come at the cost of high energy and resource consumption and great intensity of pollution emissions. Meanwhile, negative environmental externalities are becoming increasingly apparent due to the interaction between China’s energy-intensive agricultural development mode and the inefficient use of chemical inputs, as well as soil and water pollution and food safety issues associated with intensive farming and large livestock and poultry farms (Fischer et al., 2010).

Overall, agricultural problems are still serious in China (Chen, Miao & Zhu, 2021; Wang & Lu, 2020; Wang et al., 2019a). As a developing country with a large population base and shortage of arable land resources, China’s agricultural production is overly dependent on large-scale agricultural machinery, chemical fertilizers, pesticides, agricultural films, antibiotics and other consumable resources. Environmental pollution such as soil pollution of the arable land, water pollution, and air pollution is further aggregating, and ecological problems such as soil erosion, land desertification, salinization are becoming increasingly serious. The depletion of a large number of non-renewable resources is leading to a more prominent problem of resource shortage.

Long-term overdraft of agricultural resources, serious non-point source pollution in agriculture and severe ecological damage not only greatly impede the green development of agriculture, but also pose serious health hazards to residents. Excessive pesticide residues and antibiotics in agricultural products lead to food safety problems (Sun, Hu & Zhang, 2021); eutrophic water pollution is closely related to cancer incidence (Ebenstein, 2012); and heavy metals in soil pose a threat to human health (Huang et al., 2020). With the common perception of society’s environmental protection requirements and health needs, it is in urgent need to transform the traditional high-consumption and high-pollution agricultural production mode into a green agricultural development mode. In 2017, the General Office of the Central Committee of the Communist Party of China and the General Office of the State Council issued the Opinions on Innovative Institutional Mechanisms to Promote Green Development in Agriculture (The State Council, 2017), which points out that green development of agriculture means achieving sustainable agricultural development with no reduction of arable land, no over-extraction of groundwater, zero growth in the use of chemical fertilizers and pesticides, and full utilization of straw, livestock and poultry manure and agricultural films. Green development of agriculture is the driving force of green economic development and a prerequisite for achieving green behavior and sustainable ecology. Technical Guidelines for Green Agricultural Development (2018–2030) (State Council Gazette, 2018) released by the Ministry of Agriculture and Rural Affairs of China in 2018 points out that the problems of excessive pesticide residues and environmental pollution are still prominent in some regions, and the excessive use of chemical fertilizers and pesticides has led to rising agricultural production costs, reducing the competitiveness of agricultural products and the sustainability of agricultural development. Therefore, there is an urgent need to establish a technology system for green development of agriculture featuring safe and harmless agricultural inputs, economical and efficient resource utilization, environment-friendly production process, sound quality standard system, and timely monitoring. Such a technology system covers all aspects of the agricultural production behavior and consumption behavior of urban and rural residents, which not only can improve human living environment, but also has positive significance in safeguarding people’s health, improving population quality, and promoting healthy regional development with organic and pollution-free agriculture products.

Due to the external diseconomies of resource use and environmental pollution generated by agricultural production, environmental regulation as an intervention tool is an important way to achieve the green development of agriculture. With the increasing national emphasis on green development and environmental protection, China has promulgated environmental regulatory policies such as Regulation on the Administration of Pesticides, Regulation on the Protection of Basic Farmland, and Implementation Opinions of the Ministry of Agriculture on the Prevention and Control of Agricultural Non-point Source Pollution, and implemented environmental regulatory measures such as investment in environmental protection projects. However, pollution caused by agriculture is still serious at this stage. In the Yangtze River Economic Belt, one of China’s major national strategic development regions, extensive agricultural operations have led to problems such as sharp decrease of resources, ecological damage, soil erosion, environmental pollution, and frequent disasters (Shou, Du & Liu, 2019; Tian et al., 2021). Nitrogen and phosphorus pollutant concentrations in the Yangtze River Economic Belt have been slowly increasing since the 1960s, and the rate of increase has accelerated since the 1980s (Ji et al., 2020). The Yangtze River Economic Belt spans east, central and west China, including 11 provinces/municipalities, *i.e.,* Shanghai, Jiangsu, Zhejiang, Anhui, Jiangxi, Hubei, Hunan, Chongqing, Sichuan, Guizhou and Yunnan, with a total area of about 2,052,300 hm^2^, accounting for 21.37% of the country’s land area. As an important economic zone in China, its population accounted for 43.00% of the country’s population and it contributed 46.32% of the country’s GDP in 2019. In addition, the Yangtze River Economic Belt is rich in natural resources and is a major agricultural production area. In 2019, its output value was about 5.19 trillion yuan, accounting for 41.89% of the total national output value of agriculture, forestry, animal husbandry and fishery, and its grain production reached 243 million tons, accounting for 36.60% of the total national grain output (He et al., 2021). It occupies an important position in food safety and ecological security, and has a decisive impact on China’s economic development. Currently, there are significant regional differences in population health levels in the Yangtze River Economic Belt, with a low-to-high gradient pattern from the upper to lower reaches (Chen & Wu, 2020), which is not conducive to achieving the important goal of “Healthy China”. Therefore, it is imperative to accelerate the formulation and promulgation of relevant environmental regulations to effectively control environmental pollution and ensure green development. Environmental regulations enacted by the government aim not only to reduce pollution and improve environment quality, but also to improve people’s life quality from environmental aspects that are closely related to residents’ health. However, most of the existing literature either focuses on the relationship between environmental regulation and agricultural pollution and the impact of environmental regulation implementation on printing services, or analyzes the impact of agricultural production activities on health from the perspective of biochemistry, whereas the intermediate action logic and transmission chain of “environmental regulation-public health” are rarely studied.

This article attempts to explore the intrinsic relationship and influence mechanism between environmental regulation, green development of agriculture and residents’ health through the analysis of panel data of the Yangtze River Economic Belt, with a view to providing useful references for implementing environmental regulation, curbing agricultural non-point source pollution, developing green agriculture, improving residents’ health level, and ultimately, promoting healthy regional economic development.

## Literature Review

### Green development of agriculture and residents’ health

Substantial studies have focused on the negative impacts of the indiscriminate use of chemical fertilizers, pesticides and agricultural films in the agricultural production process on residents’ health. The extensive use of chemical fertilizers, pesticides, and organic fertilizers from livestock and poultry manure in agricultural production activities, as well as the discharge of domestic sewage causes surface source pollution, leading to eutrophication of nitrogen and phosphorus in water bodies (Li et al., 2020a). Nitrate nitrogen and pesticide agents in water are closely related to the incidence of cancer. Studies have shown that the deterioration of river water quality exposes people to higher economic costs of diseases and increases the incidence of digestive tract cancer (Srinivasan & Reddy, 2009; Ebenstein, 2012). In rural areas, where drinking water generally comes from surrounding water sources, heavy use of fertilizers and pesticides can easily cause pollution, thus endangering the health of rural residents (Wang et al., 2021).

The use of pesticides and fertilizers leads to the accumulation and pollution of heavy metals in agricultural soil. Upon entering the soil system, the insidious and toxic heavy metals undergo a series of physical and chemical interactions with the soil environment, resulting in their enrichment in each soil layer. Consequently, heavy metals will pose potential non-carcinogenic risks to human health through the “soil-crop-human” food chain and exposure route (direct ingestion, oral and nasal inhalation and skin contact) (Tóth et al., 2016).

Meanwhile, the use of pesticides and fungicides has a significant negative effect on human health (Atreya, 2008). Phthalate esters (PAEs) are widely used in the production of agricultural films, pesticides and fungicides (Xu, Li & Wang, 2008). As a PVC plastic product, agricultural films contain up to 20%∼50% PAEs. Studies have shown that PAEs have carcinogenic, teratogenic, mutagenic and endocrine disrupting effects. Due to their widespread use, PAEs have been widely distributed in environmental media like water, air, and soil, and accumulated in various vegetables and animals, causing damage to human health and the environment (Zhang et al., 2016).

The increasing use of chemical fertilizers and pesticides and the uncontrolled discharge of livestock manure have led to a decline in the quality of agricultural products, which is detrimental to people’s health (Min & Kong, 2016). Pesticide exposure due to pesticide application not only increases the risk of acute pesticide poisoning, but also causes chronic and long-term damage to human health (Sookhtanlou, Allahyari & Surujlal, 2022; De Silva, Albert & Jayasekara, 2017).

Therefore, the conflict between pollution from traditional agricultural development and residents’ health has become a practical problem that cannot be ignored in the development process of China. Promoting the green development of agriculture can help reduce the intensity of pesticide, chemical fertilizer, and agricultural film application, improve the utilization rate of resources such as land, water, agricultural machinery and power, and guarantee the quality of agricultural products. Accordingly, this article proposes Hypothesis 1: Green development of agriculture can improve the health level of residents.

### Environmental regulation, green development of agriculture and residents’ health

The concept of environmental regulation was first introduced by Dasgupta (1980) in the early twentieth century and has since been gradually accepted by scholars. Due to the scarcity of environmental resources and the negative externalities that may arise from their use, environmental regulation was initially defined as direct government intervention in the use of environmental resources, mainly through coercive means such as policies. Zhao, Zhu & He (2009) defined environmental regulation as a restraining force for environmental protection, with individuals or organizations as targets and tangible institutions or intangible consciousness as the form of existence.

Due to external diseconomies of resource utilization and environmental pollution generated by agricultural production, environmental regulation, as an intervention tool, is an important way to achieve green development of agriculture. In Denmark, in order to solve the problem of pesticide pollution of underground drinking water, the Danish Environmental Protection Agency issued a ban on the use of pesticides on impermeable man-made hard surfaces, and by using ultraviolet light and other alternatives to pesticides, pollution of groundwater can be reduced, thereby ensuring the safety and cleanness of drinking water in Denmark (Kristoffersen et al., 2008). Finland has also taken some environmental measures such as decreasing the use of mineral fertilizers and increasing the use of zero-tillage and reduced-tillage methods. The agri-environmental program was introduced in Finland as part of the income subsidy system, with about 85–92% of farms and 88–96% of the agricultural area committed to different program periods (Huttunen & Peltomaa, 2016).

Some scholars believe that the formulation and implementation of environmental regulatory policies are conducive to improving environmental quality and reducing pollutant emissions (Fan et al., 2021; Zhang et al., 2021; Jin, Liu & Chen, 2022; Huang & Gao, 2017; Liu et al., 2022). Environmental regulation enables enterprises to promote the reform of production technology and the innovation of environmental management technology, and the resulting economic benefits can compensate or even exceed the “crowding-out effect” and opportunity costs incurred to meet the environmental regulation constraints, which can eventually lead to “innovation compensation”. Besides, agricultural subsidies have both technology and scale effects (Guo et al., 2021; Yang & Lin, 2020). If the government adopts appropriate policy combinations, nitrate surplus can be reduced by more than 50% and phosphate surplus can even be reduced by more than 75% without significant negative impacts on China’s food self-sufficiency (Van Wesenbeeck et al., 2021).

However, some scholars hold that environmental regulation is difficult to take effect in agriculture and may even bring negative effects (Yuan & Zhu, 2015; Hu, 2017). The increased cost of environmental regulation not only increases the burden of agricultural producers, but also crowds out other investments of them, especially those in the research and development of clean production technologies with large initial investments and long lead time, which will reduce agricultural green total factor productivity in the short term. Moreover, increase in the intensity of environmental regulation will prompt agricultural producers to reduce the use of chemical fertilizers and pesticides, which will affect their profits in the short term and reduce their incentives to engage in green production (Boyd & McClelland, 1999; Cochard, Willinger & Xepapadeas, 2005). Although the fertilizer subsidy policy promotes the motivation of agricultural production, it distorts factor prices and leads to a waste of resources. In the long run, it would fall into a vicious circle of “fertilizer over-application—environmental pollution—soil structure destruction—fertility decline—fertilizer application increase”, thereby leading to excessive fertilizer application and continuous environmental degradation (Scholz & Geissler, 2018).

### Spatial spillover effects of green development of agriculture and environmental regulation on residents’ health

Pollution caused by agricultural production is a kind of harmful substance with strong spatial spillover characteristics, and geographical boundaries no longer have the general significance of division and isolation. The spatial spillover effects break the confinement and restriction of space and affect residents of neighboring areas. It has been argued in some researches that due to the spillover characteristics of environmental pollution, environmental regulation in a given region may not be beneficial to its residents. However, on the contrary, even if no regulatory policy is implemented, the region may still benefit from the environmental governance of neighboring areas. For example, carbon trading policy not only promotes the agricultural green total factor productivity in pilot areas, but also in other non-pilot areas due to technology diffusion, policy interaction and the expected behavior of economic agents (Yu et al., 2022). Meanwhile, lower environmental regulation intensity is more attractive to local governments. In the process of formulating and implementing environmental regulatory policies, “cutting corners” has become the most convenient and “economic” means for local governments to attract talents and capital, promote industrial development, and pursue higher economic benefits. That is to say, a region may share the high cost of environmental pollution with other regions while enjoying economic benefits alone, leading to the adverse phenomenon of “race to the bottom” in government environmental regulation (Feng et al., 2020). In other words, agricultural development and environmental regulation not only have an impact on residents of a certain region, but also affect residents of neighboring areas.

Therefore, this article proposes Hypothesis 2: The impact of green development of agriculture and environmental regulation on residents’ health has spatial spillover characteristics.

### Study area

As a significant food production base in China, agricultural development of the Yangtze River Economic Belt has received widespread attention, and various aspects of this issue have been addressed in previous studies. Yang et al. (2021) measured and analyzed the eco-efficiency of arable land utilization in the Yangtze River Economic Belt and noted that the lower reaches had the highest eco-efficiency, followed by the middle and upper reaches. The eco-efficiency of arable land utilization can be effectively improved through the control of carbon emissions and agricultural pollution. Using non-point source pollution as an unexpected output, Ding, Cai & Fu (2021) investigated the impact of urbanization on the efficiency of agricultural green water utilization in the Yangtze River Economic Belt. They found that urbanization accompanied by land transfer and rural labor shift promoted the efficiency of agricultural green water utilization. Other studies have mainly focused on the development of the Yangtze River Economic Belt in terms of carbon emissions from agriculture (Sun et al., 2022), high-quality agricultural development (Cui et al., 2022), and agricultural production efficiency (Pan et al., 2022). Meanwhile, as an important region with more than 40% of the country’s population, public health of the Yangtze River Economic Belt has also received academic attention. Some researches focus on the health effects of air pollution levels (Mao, Sun & Zhang, 2020; Bai et al., 2019), as well as heavy metal pollution in the Yangtze River Basin and the health risks it poses (Yan et al., 2021). As an important agricultural production area in China, the green development of agriculture in the Yangtze River Economic Belt is of great significance for ensuring national food security and achieving the goal of “Healthy China”.

### Summary

Based on the above literature review, existing studies have been fruitful in terms of theory and methodology in exploring the interaction between green development of agriculture and environmental regulation. However, more researches are still in urgent need as previous research findings are controversial and unanimous conclusions have not yet been formed on the impact of different types of environmental regulations on green development of agriculture. Moreover, most existing studies are conducted at the national level, while studies on the Yangtze River Economic Belt, the important strategic development region and food production area in China, can yield more targeted conclusions and recommendations. At the same time, effects of policy implementation not only come from within a specific region, but are also influenced by spillover effects from other regions, so it is meaningful and necessary to explore the spatial spillover effects.

As for the relationship between green development of agriculture and residents’ health, most existing studies have been conducted from the physiological perspective, and their samples are usually limited to one single experimental field. Relatively few studies have explored the impacts of green agricultural development on public health at the macro level through econometric methods.

This article, using mediating effect model, spatial econometric model, etc., investigates the effects of environmental regulation on green development of agriculture and residents’ health to reveal the intrinsic relationships and influence mechanisms of environmental regulation, green development of agriculture, and residents’ health in the Yangtze River Economic Belt.

## Data and Method

### Data

Based on data availability and statistical consistency, this article selects 11 provinces/municipalities in the Yangtze River Economic Belt as research samples, including Shanghai, Zhejiang, Jiangsu, Anhui, Jiangxi, Hubei, Hunan, Chongqing, Sichuan, Yunnan and Guizhou, and the sample period is from 2011 to 2020. Data required for command-and-control environmental regulation are obtained from the number of relevant legal provisions on PKULAW.COM (https://www.pkulaw.com/), and the rest of the variables are taken from official authoritative databases such as China Statistical Yearbook, China Agricultural Statistical Yearbook, and China Health Statistics Yearbook from 2011 to 2020. The processing of each variable is described as follows.

#### Explained variable: residents’ health is the explained variable

Residents’ health level is usually measured by mortality rate or life expectancy (Yang & Zhang, 2018; He, Fan & Zhou, 2016; Yang, Zhou & Ding, 2022; Chen et al., 2013). However, mortality rate and life expectancy are influenced by many factors, and lack accuracy in measuring the impact of agricultural development on residents’ health. This article focuses on the impact of the use of chemical fertilizers, pesticides, and agricultural films on the health of residents, since not all diseases are related to environmental pollution caused by agricultural production factors. The agricultural production environment is the source of foodborne diseases and food contamination may occur at any stage from food production to consumption (Aijuka & Buys, 2019). The risk to human health from agricultural activities is usually reflected in foodborne diseases. Drawing on Qin, Peng & Deng (2019), this article uses the number of foodborne diseases as an indicator of health status to more accurately describe the impact of environmental pollution caused by agriculture on residents’ health.

#### Explanatory variables

(1) The use of chemical fertilizers, pesticides and agricultural film in the Yangtze River Economic Belt is the first explanatory variable in this article. The impact of agriculture on residents’ health is mainly through the excessive use of chemical fertilizers and pesticides, as well as the production, use, burning and burying of agricultural films.

(2) Environmental regulation is the second explanatory variable in this article. The existing literature on environmental regulation mainly focuses on the industrial sector where data are readily available and its measurement indicators include environmental investments, treatment costs, waste emissions, etc. However, environmental regulation has rarely been applied to the agricultural field where data are difficult to obtain and measure (Shi & Yi, 2020). In addition to planting, agricultural production activities also include a series of subsequent production and processing. Agricultural production factors, such as fertilizers, pesticides and agricultural films, also involve processing and manufacturing. Therefore, drawing on the methodology of Li & Li (2012) and Zhan & Xu (2019), this article measures the intensity of market-incentive environmental regulation in terms of investments in environmental pollution control projects completed and the intensity of command-and-control environmental regulation in terms of the number of environmental provisions related to agriculture and rural areas found on PKULAW.COM (https://www.pkulaw.com/) in a given year.

#### Control variables

(1) Technological innovation. Technological innovation in agriculture can reduce agricultural pollution by promoting the technical literacy of agricultural workers and improving the technical indicators of crops, soil and agricultural materials, thereby increasing agricultural production efficiency and decreasing material consumption. Government investments in technological innovation are conducive to improving both the production technology and product quality of chemical fertilizers, pesticides, and agricultural films and the medical and health technology.

(2) Government subsidy to agriculture. Government financial subsidies to agriculture influence the scale of agricultural operation, planting structure, output level, etc. With government guiding farmers to carry out production and planting through financial subsidies, the scale effect of land-intensive industries can quickly arise in areas where planting is the primary industry, which is conducive to the implementation of comprehensive pollution control and environmental regulation. At the same time, government subsidies motivate farmers to adopt organic fertilizers, receive technical training, reduce economic costs of carrying out green agriculture, and mobilize them to green production.

(3) Per capita disposable income of rural residents. When the per capita income is low, under the premise of limited arable land areas, rural residents will increase the input of production factors such as chemical fertilizers and pesticides to promote crop output and household income, which will indirectly aggravate agricultural non-point source pollution. However, as the living standards improve, they will have higher demands on the quality of life and production environment, thus increasing awareness of environmental protection and health.

(4) Per capita disposable income of urban residents. With the improvement of income level and the popularization of health concept, the demand of urban residents for green and healthy agricultural products has greatly increased, which promotes the production and supply of green and ecological agricultural products from the demand side and boosts the green development of agriculture to a certain extent.

In order to reduce the absolute value of the data and the problems of covariance and variance of the model, the above variables are logarithmized. The variable definitions and descriptions are shown in Table 1.

**Table 1:** Variables and variable definitions.

	Variables	Symbol	Definition	Unit	Mean	Std. dev.	Max	Min
Explained variable	Residents’ health	Health	The number of foodborne diseases	People	1,263.19	1,241.39	6,621.00	6.00
Explanatory variables	Green development of agriculture	Pesticide	Pesticide consumption	10,000 tons	6.35	4.01	13.95	0.26
		Fertilizer	Chemical fertilizer consumption	10,000 tons	201.35	110.19	354.90	7.50
		Film	Agricultural plastic film consumption	10,000 tons	7.48	3.34	13.24	1.29
	Market-incentive environmental regulation	ers1	Environmental pollution control cost	100 mn RMB	21.61	17.44	81.17	3.35
	Command-and-control environmental regulation	ers2	Number of laws and regulations related to agricultural and rural environment	Item	7.24	6.41	36.00	0.00
Control variables	Technological innovation	tec	Government’s financial subsidy to agriculture	100 mn RMB	169.12	138.75	584.39	21.32
	Government assistance to agriculture	gov	Government’s financial subsidy to agriculture	100 mn RMB	629.43	263.38	1,339.36	161.54
	Per capita disposable income of rural residents	Couincome	Per capita disposable income of rural residents	10,000 RMB	3.35	1.23	7.64	1.65
	Per capita disposable income of urban residents	urbincome	Per capita disposable income of urban residents	10,000 RMB	1.37	0.65	3.49	0.45

### Methods

Fixed effects model is used to analyze the effect of environmental regulation on the health level of residents, and Eq. (1) is constructed as follows, where i represents province, t represents year, *λ*_i_ is the fixed effect of each province, ∑X_it_ is the control variable, and *ɛ*_1it_ is the error term.

(1) \begin{eqnarray*}{\text{Health}}_{\mathrm{it}}={\alpha }_{0}+{\alpha }_{1}{\mathrm{pol}}_{it}+\phi \sum {X}_{it}+{}_{1it}\end{eqnarray*}

Health_it_ is the explained variable, denoting the health level of residents in province i in year t. Pol_*it*_represents the source of agricultural pollution, *i.e.,* the amount of chemical fertilizers, pesticides, and agricultural films used, in province i in year t. In order to facilitate the interpretation of the statistical meaning and to smooth out data fluctuations, the above variables are logarithmically processed.

Fertility is produced by the dilution and decomposition of chemical fertilizers in the soil. However, excessive application of chemical fertilizers will lead to insufficient decomposition and inefficient utilization, which can disrupt the soil acid–base balance and pollute the environment. Moreover, the decomposition of chemical fertilizers in soil is persistent and gradually decreases over time. Due to the persistent and insufficient decomposition, there is a time lag in agricultural non-point source pollution caused by the excessive application of chemical fertilizers (Zainuddin et al., 2022). This is also true for the use of pesticides. Therefore, in this article, based on Eq. (1) an agricultural pollution lag term is introduced as an explanatory variable in Eq. (2) to verify the impact of non-point source pollution caused by previous excessive application of chemical fertilizers and pesticides on the health level of residents in a given year.

(2) \begin{eqnarray*}{\text{Health}}_{\mathrm{it}}={\alpha }_{0}+{\alpha }_{1}\mathrm{ers}i{t}_{}+\beta {\mathrm{pol}}_{i,t-1}+\phi \sum {X}_{it}+{}_{1it}\end{eqnarray*}

Environmental regulation cannot directly affect the health of residents; rather, they tend to indirectly affect public health through the formulation and implementation of policies. The objects of environmental regulation are all kinds of pollution emissions, and the effects are measured by the changes of these emissions and their impact on residents’ health over a certain period of time. Therefore, this article introduces a mediating effect model to explore the impact path of environmental regulation and agricultural pollution sources on residents’ health level. The two-step regression method is used to construct the mediating effect model, as shown in Eq. (3) and Eq. (4) (Wen et al., 2004):

(3) \begin{eqnarray*}{\mathrm{Pol}}_{\mathrm{it}}={\alpha }_{0}+{\alpha }_{1}\text{ears}i{t}_{}+{e}_{1}\end{eqnarray*}

(4) \begin{eqnarray*}{\text{Health}}_{\mathrm{it}}={\alpha }_{0}+{\alpha }_{1}\mathrm{ers}i{t}_{}+{\alpha }_{2}\mathrm{pol}i{t}_{}+\phi \sum {X}_{it}+{e}_{2}\end{eqnarray*}

The steps of mediating effect test are as follows: first, the regression coefficient of explanatory variables on mediating variables is tested to see if it is significant. If significant, Eq. (4) is tested again. If the regression coefficient of the mediating variables in Eq. (4) is also significant, a partial mediating effect can be identified. If the regression coefficient of the explanatory variables in Eq. (3) is significant, but the coefficient of explanatory variables is not significant in Eq. (4), then, a complete mediating effect can be identified.

While classical statistics requires data to satisfy independence, the study of spatial data is becoming more common with the increasing maturity of geographic information technology, which considers the spatial correlation and spatial heterogeneity of the data. Tobler (1979) proposed the first law of geography: everything is spatially correlated, and things that are close together have greater spatial correlation than those far away. Cliff & Ord (1979) introduced the concept of spatial autocorrelation and showed the nature of spatial weighting matrices. Anselin (1988) proposed a generalized spatial model that encompassed the study of spatial effects and the autocorrelation of error terms, and developed a spatial correlation characteristics model and a spatial autoregressive model. As the research progressed, the spatial Dubin model was proposed, which takes the spatial lags of the dependent and independent variables into account and can better explain spatial relationships.

In order to verify the spatial spillover effects of the level of agricultural green development and the intensity of environmental regulation, a spatial Dubin model is constructed in this article, drawing on Song & Cui (2019), as shown in Eq. (5):

(5) \begin{eqnarray*}\mathrm{Y }\,=\,\delta \mathrm{WY }\,+\,\alpha {\mathrm{l}}_{\mathrm{N}}\,+\,\mathrm{X}\beta \,+\,\mathrm{WX}\theta \,+\,\end{eqnarray*}

Where *Y* is an *N* × 1 order vector consisting of the observed value of the explained variables of each unit( *i* = 1,…,N) in the sample. According to the research construction in this article, *Y* is composed of the number of patients with foodborne diseases in the 11 provinces/municipalities in the Yangtze River Economic Belt in China. *X* is an *N* × *K* order explanatory variable matri6x. *W* represents the spatial weight matrix, and *WY* represents the spatial interaction between the explanatory variables, *i.e.,* the average impact of the explanatory variables in the geographically proximate regions on the explanatory variables in the region. The degree and direction of the impact are represented by *δ*. *WXθ* represents the spatial interaction between explanatory variables, *i.e.,* the average impact of the explanatory variables in geographically proximate regions on the explained variables in the region. The degree and direction of the impact are represented by *θ* . *l*_*N*_ is an *N* × 1 order unit vector, which is related to the estimated constant term parameter $\alpha .={ \left( {}_{1},\ldots {}_{i} \right) }^{T}$ is the vector of the interference term, where, for all *i*, *ɛ*_*i*_ isassumed to be independently and identically distributed, with a mean value of zero and variance of *σ*^2^.

## Results and Discussion

### Regression results and analysis

First, in order to avoid the problem of multicollinearity among variables from biasing the regression results, a multicollinearity test is performed before the regression, with variance inflation factor (VIF) used for the test. It is found that all VIF values are less than 10, indicating that there is no serious multicollinearity problem in the model.

Fixed effect and random effect are estimated and analyzed for Eq. (1), and according to the Hausman test results (see Table 2), the fixed effect is superior to the random effect. Therefore, this article uses fixed effect for regression analysis. As shown in Table 3, the estimation results of Eq. (1) show that each unit increase in chemical fertilizer use results in 0.615 cases of foodborne illnesses and it is significant at the 5% confidence level. This indicates that increasing the use of chemical fertilizers will have a negative impact on resident’ health.

**Table 2:** Results of the Hausman test.

Independent variable	Chi2	Prob
Pesticide	14.66	0.00119
Fertilizer	15.06	0.0101
Film	15.61	0.0081

**Table 3:** Regression results of green development level of agriculture.

	(1)	(2)	(3)	(4)	(5)	(6)	(7)	(8)	(9)
	Health	Health	Health	Health	Health	Health	Health	Health	Health
fertilizer	0.615^**^								
	(0.251)								
tec	1.269	0.982	0.409	1.206	1.104	0.566	1.182	1.108	0.452
	(0.761)	(0.541)	(0.323)	(0.702)	(0.621)	(0.325)	(0.694)	(0.634)	(0.333)
gov	−0.389	−0.0184	0.0918	−0.0992	0.116	0.116	−0.130	−0.0147	0.122
	(0.296)	(0.242)	(0.185)	(0.355)	(0.271)	(0.187)	(0.416)	(0.300)	(0.177)
urbincome	4.162	3.860	2.688	4.797	3.212	1.697	4.731	3.351	4.239
	(2.824)	(2.724)	(3.011)	(3.018)	(2.734)	(3.030)	(3.329)	(3.482)	(4.150)
couincome	−3.456	−2.793	−1.357	−3.894	−2.324	−0.442	−4.213	−2.940	−3.168
	(2.651)	(2.360)	(2.246)	(2.961)	(2.600)	(2.247)	(3.270)	(3.219)	(3.332)
L.fertilizer		2.063^***^							
		(0.112)							
L2.fertilizer			1.411^***^						
			(0.171)						
pesticide				0.953					
				(0.791)					
L.pesticide					1.270^*^				
					(0.698)				
L2.pesticide						1.087^**^			
						(0.441)			
film							0.896		
							(1.031)		
L.film								1.169	
								(1.026)	
L2.film									1.866^**^
									(0.812)
_cons	−4.068	−12.15^**^	−5.788^*^	−4.586	−4.530	−0.351	−4.402	−3.998	−3.957
	(5.469)	(3.834)	(2.413)	(7.461)	(6.283)	(3.283)	(7.909)	(6.951)	(4.517)
*N*	110	99	88	110	99	88	110	99	88
*R* ^2^	0.522	0.610	0.545	0.520	0.509	0.502	0.515	0.501	0.515
adj. *R*^2^	0.499	0.589	0.518	0.497	0.482	0.472	0.492	0.474	0.486

**Notes.**

Standard errors in parentheses.

* *p* < 0.1

** *p* < 0.05

*** *p* < 0.01

The regression results of Eq. (2) show that the impact of chemical fertilizers applied in the previous year on residents’ health is significant at the 1% confidence level; the impact of pesticides applied in the previous year is significant at the 10% confidence level of; and agricultural films have no significant impact on residents’ health. As for those applied in the previous two years, chemical fertilizers have a significant impact on residents’ health at the 1% confidence level, while both pesticides and agricultural films have a significant impact on residents’ health at the 5% confidence level. The lagging of chemical fertilizer, pesticide and agricultural film use have a more significant impact on the number of patients with foodborne diseases. In addition, the impact of chemical fertilizer use in the previous year on residents’ health is about 3.4 times that of fertilizer use in the current year, and the chemical fertilizer use in the previous two years on residents’ health is about 2.3 times that of fertilizer use in the current year. Similarly, both residual pesticides and agricultural films have a greater impact on residents’ health than those used in the current year. It is clear that there is a certain time lag in the pollution caused by excessive application of chemical fertilizers and pesticides due to inadequate and persistent decomposition. The impact of agricultural films on health is mainly caused by the disposal of waste films, *i.e.,* discarding, burning, burying (Xue et al., 2021). Since the average life span of agricultural films is usually about 2-3 years, the use of agricultural films has no significant impact on the health of residents in the short term. However, the main components of agricultural film materials are polymer compounds, which are difficult to decompose under natural conditions. As a result, their long-term retention in the arable land will affect the soil permeability, impede water and fertilizer transport, and damage the soil structure (Li et al., 2020b). Then, to ensure crop yields, producers will increase the use of chemical fertilizers, creating a vicious circle and posing health risks to residents.

The above findings confirm Hypothesis 1: the use of chemical fertilizers, pesticides and agricultural films in agricultural production activities have negative impacts on the health level of residents.

### Mediating effect analysis

In order to analyze the effect of environmental regulation mechanism and agriculture on residents’ health, green development of agriculture is used as a mediating variable for mediating effect analysis. As shown in Table 4, the original hypothesis is rejected by the Sobel test, indicating that the mediating effect is significant.

**Table 4:** Regression results of the mediating effect.

	(1)	(2)	(3)	(4)	(5)
	Health	Health	Health	Health	Health
Fertilizer	1.087^***^	1.049^***^			
	(0.165)	(0.170)			
ers1	0.239^*^		0.129		0.00622
	(0.110)		(0.110)		(0.110)
tec	−0.276	−0.108	−0.120	−0.0334	−0.181
	(0.189)	(0.175)	(0.188)	(0.173)	(0.183)
gov	−0.365	−0.241	0.188	0.214	−0.247
	(0.313)	(0.317)	(0.249)	(0.250)	(0.280)
urbincome	11.17^***^	10.67^***^	11.93^***^	11.82^***^	7.770^***^
	(1.186)	(1.194)	(1.287)	(1.297)	(0.843)
couincome	−6.773^***^	−6.626^***^	−8.272^***^	−8.264^***^	−4.767^***^
	(0.801)	(0.816)	(0.939)	(0.948)	(0.687)
ers2		0.0400		0.0300	
		(0.0871)		(0.0863)	
pesticide			0.853^***^	0.857^***^	
			(0.131)	(0.133)	
film					1.704^***^
					(0.238)
_cons	−7.209^***^	−7.479^***^	−7.556^***^	−7.704^***^	−2.065
	(1.245)	(1.266)	(1.251)	(1.252)	(1.410)
*N*	110	110	110	110	110
*R* ^2^	0.665	0.651	0.663	0.659	0.682
adj. *R*^2^	0.646	0.630	0.643	0.639	0.663

**Notes.**

Standard errors in parentheses

* *p* < 0.1

** *p* < 0.05

*** *p* < 0.01

Market-incentive environmental regulation has a complete mediating effect on pesticides and agricultural films, while command-and-control environmental regulation has a complete mediating effect on chemical fertilizers, pesticides, and agricultural films, and the regression coefficients are all positive. This suggests that intensifying environmental regulation may increase the use of chemical fertilizers, pesticides and agricultural film, thus leading to a decrease in residents’ health level.

Possible reasons why the empirical results are contrary to the original intention of environmental regulation are as follows. Firstly, it has been pointed out in some studies that there is a non-linear relationship between environmental regulation and green development of agriculture, *i.e.,* first inhibiting and then promoting  (Zheng et al., 2023; Wang & Shen, 2016). Current environmental regulation in the field of agriculture is characterized by low application, weak intensity, lack of punishment measures, and little or no policy subsidy for green agriculture. It may not have crossed the tipping point, and not yet possible to promote green development of agriculture in the short term. For agricultural enterprises, to comply with environmental protection standards and norms, they need to purchase pollution control equipment and invest in green innovation in the process of pollution control, which increases production costs while reduces non-green R&D expenditures and crowds out production investments. Alternatively, out of pursuit of profits, enterprises are more willing to invest in production expansion, because profits gained from it can offset the costs of environmental regulation, thereby weakening their motivation to green production. Previous researches have also pointed out that environmental regulation has negative effects on green agricultural development in China. Since 2000, the cost effect of environmental regulation on green development of agriculture has been greater than the “innovation compensation” effect, which is not sufficient to offset the negative impacts of governance costs (Ma & Tan, 2021). Secondly, small holder-based family business is the main form of agricultural operation in China, accounting for more than 98% of agricultural operation entities, 90% of the total agricultural employees, and 70% of the total arable land (Wang et al., 2015). If solely relying on government’s regulatory means to achieve green development of agriculture, the cost would be extremely high. Due to the lack of relevant environmental regulation, coupled with the severe shortage of environmental resource allocation such as public environmental facilities and services in rural areas, it is difficult to raise farmers’ awareness and positive behavior for pollution prevention and control, and the production and living behaviors of farmers tend to further aggravate agricultural pollution (Wang et al., 2019b). In addition, the environmental regulation process of agricultural pollution prevention and control is slow and passive. Corresponding regulatory measures are introduced only when a certain type of agricultural pollution is prominent. What is worse, some specific agricultural pollution prevention and control measures or projects are difficult to take effect due to the unreasonable benefit mechanism or the lack of incentive mechanism. When the ecological compensation mechanism for agricultural pollution prevention and control is missing or imperfect, stakeholders whose interests are directly damaged will be financially motivated to abandon environment-friendly behaviors.

### Spatial dubin model

Spatial correlation tests, including LM-Lag, robust LM-Lag, LM-Error, and robust LM-Error tests, are performed on the sample data to determine the type of spatial effects and to select the model form. The results in Table 5 show that there are tests rejecting the original hypothesis for both spatial errors and spatial lag, indicating that spatial econometric analysis should be performed.

**Table 5:** LM test results.

		Statistic	*p*-value
pesticide	LM-Error	6.336	0.012
	Robust LM-Error	11.467	0.001
	LM-Lag	1.485	0.223
	Robust LM-Lag	6.616	0.010
fertilizer	LM-Error	9.801	0.002
	Robust LM-Error	14.026	0.000
	LM-Lag	0.458	0.498
	Robust LM-Lag	4.683	0.030
film	LM-Error	16.835	0.000
	Robust LM-Error	20.137	0.000
	LM-Lag	0.077	0.781
	Robust LM-Lag	3.379	0.066
ers1	LM-Error	3.026	0.082
	Robust LM-Error	9.762	0.002
	LM-Lag	8.820	0.003
	Robust LM-Lag	15.556	0.000
ers2	LM-Error	1.364	0.243
	Robust LM-Error	5.221	0.022
	LM-Lag	6.388	0.011
	Robust LM-Lag	10.245	0.001

The SDM model in spatial econometrics is used to empirically analyze the relevant data of the 11 provinces/municipalities in the Yangtze River Economic Belt in China from 2011 to 2020. After a Hausman test(see Table 6 for the results), this article selects the random effect model and results are presented in Table 7.

**Table 6:** Results of the Hausman test for SDM.

Independent variable	Chi2	Prob
pesticide	0.26	0.6102
fertilizer	0.00	0.9680
film	1.08	0.2998
ers1	−0.29	/
ers2	−3.36	/

**Table 7:** Regression results of spatial Dubin model.

	(1)	(2)	(3)	(4)	(5)
	Health	Health	Health	Health	Health
Main					
fertilizer	0.220				
	(0.225)				
pesticide		−0.0318			
		(0.269)			
film			1.218^***^		
			(0.360)		
ers1				0.256^*^	
				(0.134)	
ers3					−0.0735
					(0.0936)
_cons	10.23^***^	8.378^***^	1.518	2.612^***^	2.711^***^
	(3.724)	(1.837)	(2.565)	(0.987)	(0.720)
Wx					
pesticide fertilizer	−1.589^***^				
	(0.606)				
pesticide		−2.381^***^			
		(0.641)			
film			−0.389		
			(1.154)		
ers1				−0.102	
				(0.288)	
ers2					0.523^**^
					(0.214)
Spatial					
rho	0.474^***^	0.247^*^	0.542^***^	0.550^***^	0.486^***^
	(0.106)	(0.147)	(0.0955)	(0.0956)	(0.104)
Variance					
lgt_theta	−1.089^***^	−1.402^***^	−0.603^*^	−1.221^***^	−1.277^***^
	(0.314)	(0.304)	(0.350)	(0.294)	(0.290)
sigma2_e	0.429^***^	0.397^***^	0.442^***^	0.435^***^	0.428^***^
	(0.0620)	(0.0568)	(0.0643)	(0.0634)	(0.0622)
*N*	110	110	110	110	110
*R* ^2^	0.166	0.090	0.333	0.021	0.032
adj. *R*^2^					

**Notes.**

Standard errors in parentheses.

* *p* < 0.1

** *p* < 0.05

*** *p* < 0.01

The concepts of direct effects and indirect effects are introduced here: if a change of a particular explanatory variable in a unit changes not only the explained variable in its unit, but also the explanatory variables in other units, then, the change within the unit is called direct effects, and the change in other units is called indirect effects. According to the concept, it is not difficult to find that indirect effects is in fact spatial spillover effects. Therefore, indirect effects estimated by the explanatory variable rather than the variable estimation coefficient should be used to test whether spatial spillover effects exist. Based on this, the data of direct effects, indirect effects and total effects in Table 8 are obtained.

**Table 8:** Direct, indirect and total effects of spatial regression.

	Direct effects	Indirect effects	Total effects
fertilizer	0.0776	−2.546^**^	−2.468^**^
	(0.257)	(1.068)	(1.233)
pesticide	−0.126	−2.984^***^	−3.110^***^
	(0.263)	(0.600)	(0.662)
film	1.280^**^	0.738	2.017
	(0.516)	(2.747)	(3.197)
ers1	0.270^*^	0.138	0.409
	(0.146)	(0.579)	(0.641)
ers2	−0.0122	0.944^**^	0.932^**^
	(0.0998)	(0.382)	(0.423)

**Notes.**

Standard errors in parentheses

* *p* < 0.1

** *p* < 0.05

*** *p* < 0.01

For further analysis, the two variables, green development of agriculture and environmental regulation, are explored respectively in this article. (1) Spatial spillover effects of green development of agriculture on residents’ health.

According to the regression results, the indirect effects of agricultural films are not significant. Pollution caused by the use of agricultural films in agricultural production activities is mainly constrained in the soil, which is unlikely to form pollution spillover.

However, chemical fertilizers and pesticides have high negative spillover effects. The indirect effects of chemical fertilizers and pesticides are −2.546 and −2.984, respectively, indicating that each increase in the use of chemical fertilizers and pesticides in neighboring provinces/municipalities leads to a decrease in the number of foodborne diseases in a certain province/municipality. On the one hand, the increase of agricultural pollution emissions in neighboring provinces/municipalities will arouse vigilance, so more efforts will be put into pollution prevention. On the other hand, due to geographical conditions and urban development, areas that are not suitable for agricultural production activities or have a high urbanization rate will purchase agricultural products from other provinces/municipalities to meet local production and living needs. For example, in Shanghai, a first-tier city with an urbanization rate close to 90%, only 40% of vegetables consumed come from local supply, with the remaining 60% rely on neighboring provinces (Shanghai Municipal People’s Government, 2021). While some regions undertake more agricultural production activities and use more chemical fertilizers and pesticides to increase crop yields, other regions purchase finished products rather than produce locally to reduce the input of production factors such as chemical fertilizers and pesticides, thus lowering the health risks associated with agricultural production activities. (2) Spatial spillover effects of environmental regulation on residents’ health.

According to the regression results of the spatial Dubin model, the direct effects of market-incentive environmental regulation is 0.27 at the 10% confidence level, indicating that every additional 27 million yuan spent on pollution project governance leads to an increase of one foodborne disease patient. Pollution caused by production factors such as chemical fertilizers and pesticides used in agricultural production activities harm human health through various ways. It is difficult to eliminate the negative impact of such pollution on human health by end-of-pipe prevention and control like pollution project governance. At the same time, the rising costs of pollution control will induce the expansion of hidden economic activities (Yu & Gao, 2015), increase the difficulty of government supervision, and to a certain extent weaken the policy effect of environmental regulation, thus aggravating pollution and causing damage to the health of residents. In contrast, the indirect effects of market-incentive environmental regulation are not significant. Pollution control has a greater impact on local residents.

The direct effects of the command-and-control environmental regulation are −0.0122, which indicates that locally enacted legal documents can prevent the deterioration of residents’ health caused by the heavy use of chemical fertilizers, pesticides and agricultural films in agricultural production to a certain extent. However, at the 5% confidence level, the indirect effects are 0.944, indicating that although residents benefit from the locally enacted laws and regulatory policies, their health is negatively affected by those enacted in neighboring areas. Due to the scarcity of agricultural arable land resources, there is a conflict between the demand for increasing crop production and the need for environmental protection. The adoption of high-intensity environmental regulation and stringent pollutant emission standards by the government may produce a pollution refuge effect (Choi, 2022), leading to the transfer of pollution emissions to neighboring areas with lax environmental regulation and endangering the health of residents there. This confirms the findings of Peng (2020) and Feng et al. (2020) that environmental regulation in neighboring areas inhibits green production. Increasing in the intensity of environmental regulation in neighboring areas will lead to an increase in pollution, and areas with lax environmental regulation will become pollution shelters. The spatial spillover effects offset the environmental improvement effects of local environmental regulation.

The above findings validate Hypothesis 2: green development of agriculture and command-and-control environmental regulation have significant spatial spillover characteristics. The health level of residents is not only related to the local use of chemical fertilizers and pesticides and local environmental regulation, but is also affected by those in neighboring areas.

### Robustness test

To strengthen the reliability of the regression results, robustness tests are conducted in the following two ways. First, since the number of hospitalizations is an indicator for residents’ health level, it is used to replace the original explanatory variables for each region. Second, government health expenditure affects the level of health care in a region, so it is added as a control variable for regression. As presented in Table 9, the regression results show that except for data differences in the regression coefficients, the results are similar to previous results, which proves the robustness of the results.

**Table 9:** Robustness test.

	(1)	(2)	(3)	(4)	(5)	(6)	(7)	(8)	(9)
	Hospitalizations	Hospitalizations	Hospitalizations	Hospitalizations	Hospitalizations	Hospitalizations	Hospitalizations	Hospitalizations	Hospitalizations
pesticide	0.249^**^								
	(0.0798)								
tec	0.0651	0.0474	−0.0495	0.0808	0.0462	−0.0505	0.0653	0.0558	−0.0699
	(0.0795)	(0.0765)	(0.0642)	(0.0738)	(0.0787)	(0.0643)	(0.0851)	(0.0745)	(0.0615)
gov	0.126	0.106	0.136	0.0381	0.0652	0.122	0.112	0.0799	0.134
	(0.0927)	(0.108)	(0.0803)	(0.0814)	(0.101)	(0.0731)	(0.109)	(0.116)	(0.0831)
urbincome	0.223	0.487	3.471^***^	0.0651	0.390	3.327^***^	0.144	0.405	3.932^***^
	(0.744)	(0.629)	(0.642)	(0.673)	(0.548)	(0.685)	(0.799)	(0.611)	(0.648)
couincome	0.859	0.681	−1.923^**^	0.916	0.648	−1.774^*^	0.872	0.694	−2.430^**^
	(0.636)	(0.586)	(0.565)	(0.594)	(0.516)	(0.587)	(0.694)	(0.568)	(0.549)
medical	−0.314	−0.394^*^	−0.366^*^	−0.255	−0.310^**^	−0.429^**^	−0.352	−0.407^*^	−0.361^*^
	(0.157)	(0.143)	(0.117)	(0.168)	(0.0894)	(0.110)	(0.179)	(0.135)	(0.122)
L.pesticide		0.184^**^							
		(0.0595)							
L2.pesticide			0.217^**^						
			(0.0754)						
fertilizer				0.199^***^					
				(0.0494)					
L.fertilizer					0.157^***^				
					(0.0210)				
L2.fertilizer						0.108^***^			
						(0.0334)			
film							0.174		
							(0.128)		
L.film								0.0937	
								(0.0943)	
L2.film									0.349^***^
									(0.0816)
_cons	6.434^***^	6.974^***^	4.161^***^	6.097^**^	6.332^***^	4.573^***^	6.889^***^	7.372^***^	3.509^***^
	(0.928)	(1.133)	(0.905)	(1.335)	(0.887)	(0.884)	(1.224)	(1.152)	(0.811)
N	110	99	88	110	99	88	110	99	88
R2	0.923	0.912	0.920	0.927	0.915	0.917	0.918	0.908	0.925
adj. R2	0.919	0.906	0.914	0.923	0.909	0.911	0.914	0.902	0.919

**Notes.**

Standard errors in parentheses

* *p* < 0.05

** *p* < 0.01

*** *p* < 0.001

## Conclusions and Recommendations

Using panel data from the 11 provinces/municipalities in the Yangtze River Economic Belt in China from 2011 to 2020, this article systematically analyzes the relationship between green development of agriculture and residents’ health, the mediating effects of environmental regulation on green development of agriculture and residents’ health, and the spatial spillover effects of green development of agriculture and environmental regulation. The main findings are as follows:

First, the use of chemical fertilizers, pesticides and agricultural films is harmful to the health of residents, and it is positively correlated to environmental pollution and residents’ health. Moreover, the negative impacts usually lag behind due to the difficulty of full decomposition of chemical fertilizers and pesticides in the soil, as well as the improper treatment of waste agricultural films. The health effects of the residual chemical fertilizers, pesticides and agricultural films are even more serious.

Second, there is a mediating effect between environmental regulation and green development of agriculture and residents’ health. At this stage, the cost effect of environmental regulation in agriculture is greater than the innovation compensation. The insufficient strength of environmental regulation, the lack of green awareness among farmers, and the lag of policies have led to the inability of current environmental regulation to promote green development of agriculture.

Third, chemical fertilizers, pesticides, and environmental regulation has spatial spillover effects on the health of residents. The level of green development of agriculture and environmental regulation not only affect the health of local residents, but also have great impacts on neighboring areas.

Based on the above findings, this article proposes the following policy recommendations:

Firstly, a regional coordination mechanism for environmental regulation should be established; policies and target planning for environmental regulation should be formulated in a scientific and rational manner; and environmental regulatory policies should be implemented and monitored comprehensively and efficiently. The spatial spillover effects of chemical fertilizers, pesticides and environmental regulation on residents’ health should be given full consideration. In addition, an effective exchange and communication mechanism with governments in neighboring areas should be established in order to increase their participation in the formulation and implementation of environmental regulatory policies. This also helps to resolve possible policy contradictions and conflicts of interest among governments and avoid the singularity of decision makers and the closedness of policy visions in environmental regulation decision-making.

Secondly, an environmental push-back mechanism should be established to guide agricultural enterprises and farmers to shift to green production. Findings of the empirical analysis show that the implementation of environmental regulation does not dramatically promote the development of green agriculture, mainly due to the low intensity of environmental regulation at this stage and the lack of substantial penalties and sufficient subsidies for green agriculture. As a result, driven by interests, enterprises and farmers tend to reduce green production. Therefore, practical policies should be formulated to regulate the production behavior of enterprises and farmers. Strict emission standards should be set to strengthen constraints on the emission of pollutants through “pollution source reduction” and “end-of-pipe pollution control”. Moreover, the environmental push-back mechanism can put environmental responsibility into practice, promoting enterprises and farmers to gradually move to green production.

Thirdly, publicity and education on green production in agriculture should be carried out to improve the health of residents and to increase farmers’ knowledge and understanding of green production practices. Farmers should comply with the regulatory requirements, and adjust and regulate their production behavior in the direction of greening. At the same time, farmers who adopt green planting should be given financial subsidies or material incentives to reduce the transaction costs of their participation in green production. When farmers are guaranteed to receive minimum compensation or income, more stable economic expectations can be developed, thus motivating them to participate in green production.

## Limitations and Future Work

First, the use of machinery in agricultural production activities and agricultural irrigation may also cause non-point source pollution, which in turn may harm the health of residents. However, this study only investigated the use of chemical fertilizers, pesticides and agricultural films, without considering other factors related to the green development of agriculture. Second, except for mediating effects and spatial spillover effects, other possible influencing mechanisms on the transmission path of “environmental regulation—green development of agriculture” were not analyzed, which should be further explored in future studies.

##  Supplemental Information

10.7717/peerj.15717/supp-1 Data S1 Data

## Additional Information and Declarations

### Data Availability

The following information was supplied regarding data availability: The raw measurements are available in the Supplemental File.
